# Retroperitoneal Masses Decoded: A Practical Multidisciplinary Diagnostic and Surgical Strategy Integrating Radiology and Pathology

**DOI:** 10.7759/cureus.104707

**Published:** 2026-03-05

**Authors:** Sakshi Jaiswal, Umang K Agrawal, Deepanshu Singh

**Affiliations:** 1 General Surgery, ESIC Medical College and Hospital, Varanasi, IND; 2 Pathology, Rajendra Institute of Medical Sciences, Ranchi, IND

**Keywords:** biopsy strategy, immunohistochemistry, leiomyosarcoma, liposarcoma, lymphoma, retroperitoneal mass, retroperitoneal sarcoma

## Abstract

Retroperitoneal masses comprise a heterogeneous group of benign and malignant entities originating from mesenchymal, lymphoid, neurogenic, germ cell, and metastatic tissues. Because of the large potential space of the retroperitoneum, tumors frequently attain significant size before detection and therefore present diagnostic and therapeutic challenges. Management varies widely depending on tumor biology; consequently, inappropriate surgical intervention may lead to major morbidity without therapeutic benefit.

This narrative review provides a clinically oriented diagnostic and management framework integrating radiologic assessment, biopsy strategy, operative planning, and pathological interpretation. Retroperitoneal sarcomas require complete macroscopic resection, whereas lymphoma and germ cell tumors are primarily treated medically. Multidisciplinary coordination between surgeons, radiologists, and pathologists is therefore essential to avoid mismanagement and improve patient outcomes.

## Introduction and background

The retroperitoneum is a complex anatomical compartment extending from the diaphragm superiorly to the pelvic brim inferiorly. It contains the abdominal aorta, inferior vena cava, extensive lymphatic networks, sympathetic chains, kidneys, adrenal glands, pancreas, ureters, and portions of the colon and duodenum. Because the retroperitoneal space consists predominantly of loose areolar tissue, tumors may expand considerably before producing symptoms, frequently reaching very large sizes at presentation [1,2].

Retroperitoneal masses constitute a diagnostic category rather than a single disease entity. These lesions arise from diverse tissue origins including mesenchymal, lymphoid, neurogenic, germ cell, and metastatic sources [3]. Epidemiologic analyses demonstrate that a majority of primary retroperitoneal tumors are malignant, most commonly sarcomas [4]. However, lymphoma and germ cell tumors remain critical differentials because their management strategies differ fundamentally from those of sarcoma [5].

Historically, exploratory laparotomy was often undertaken to establish diagnosis. However, advances in cross-sectional imaging, image-guided biopsy techniques, immunohistochemistry, and molecular diagnostics have shifted management toward histology-driven therapy [6]. The modern approach emphasizes accurate diagnostic triage prior to intervention. Consequently, the surgeon’s role begins not in the operating theater but in determining the biological nature of the mass.

## Review

Epidemiology and classification

Primary retroperitoneal tumors are rare neoplasms representing a very small fraction of overall oncologic disease burden, accounting for less than 0.2% of all malignancies and approximately 10-15% of soft tissue sarcomas [7]. Their rarity explains the absence of large randomized trials and the dependence on multi-institutional databases and consensus guidelines for management recommendations. Most patients present in the fifth to seventh decades of life, although certain tumor types such as germ cell tumors occur predominantly in younger individuals [8].

Sarcomas constitute nearly 70-80% of primary malignant retroperitoneal tumors, with liposarcoma and leiomyosarcoma forming the dominant histologic subtypes [9]. The retroperitoneum uniquely favors these tumors because of the presence of abundant mesenchymal tissue and the ability of slow-growing malignancies to expand without early detection. Other less frequent sarcomas include undifferentiated pleomorphic sarcoma, malignant peripheral nerve sheath tumor, and solitary fibrous tumor. In contrast, lymphoma and germ cell tumors originate from lymphoid tissue or embryologic remnants and therefore exhibit systemic rather than localized biological behavior [10].

The World Health Organization classification integrates histologic morphology with molecular genetic alterations to define tumor subtypes and prognostic categories. Molecular diagnostics have become particularly important in retroperitoneal tumors because imaging and morphology may overlap. Amplification of the MDM2 gene confirms well-differentiated and dedifferentiated liposarcoma and distinguishes it from benign lipomatous tumors, preventing misdiagnosis and inappropriate management [11]. Similarly, recognition of specific histologic patterns allows differentiation between indolent tumors that primarily recur locally and aggressive tumors with metastatic potential.

From a clinical perspective, classification into three biological categories is most useful: localized mesenchymal tumors (sarcoma), systemic hematologic malignancies (lymphoma), and chemosensitive embryologic tumors (germ cell tumors). This biologically oriented grouping determines treatment strategy and prevents unnecessary surgical intervention in systemic diseases [9]. For this reason, retroperitoneal masses should be approached not as a single entity but as a diagnostic framework in which accurate categorization precedes therapy.

Another important epidemiologic principle is centralization of care. Studies demonstrate improved outcomes when patients with retroperitoneal sarcoma are treated in specialized centers with multidisciplinary expertise, reflecting planned surgical strategy and coordinated oncologic management [12]. Therefore, early referral to experienced centers is considered part of optimal initial management rather than a secondary consideration.

Clinical presentation

Retroperitoneal tumors frequently remain clinically silent for prolonged periods because the retroperitoneal space allows gradual expansion without early compression of hollow viscera [7]. Unlike intraperitoneal malignancies that produce obstruction early in their course, retroperitoneal lesions displace adjacent organs rather than invade them initially. Consequently, patients often present only after tumors reach a substantial size, commonly exceeding 20-30 cm in maximal dimension [2]. Many lesions are discovered incidentally during imaging performed for unrelated complaints, reflecting the insidious nature of disease progression.

The most common presenting symptoms are vague and nonspecific. Patients typically report dull abdominal discomfort, early satiety, back pain, or progressive abdominal distension. Palpable abdominal mass may be noted in thin individuals but is often absent in obese patients. Because these symptoms mimic benign gastrointestinal conditions, diagnostic delay is common.

Certain clinical patterns, however, provide important diagnostic clues. Constitutional symptoms such as fever, night sweats, and unexplained weight loss strongly suggest lymphoma and reflect systemic inflammatory activation [13]. Episodic hypertension, palpitations, headache, and diaphoresis raise suspicion for catecholamine-secreting paraganglioma and require biochemical evaluation before any invasive procedure to prevent perioperative hemodynamic instability [14]. Young males presenting with a midline retroperitoneal mass should be evaluated for germ cell tumor with measurement of serum tumor markers, including alpha-fetoprotein and beta-human chorionic gonadotropin [8].

Neurological manifestations may occur when tumors involve the psoas muscle or lumbar plexus, producing radicular pain, paresthesia, or lower limb weakness [15]. Inferior vena cava compression may result in lower-extremity edema, while ureteric displacement may produce hydronephrosis and flank pain. These secondary effects reflect mass effect rather than direct organ invasion.

Although individual symptoms lack specificity, the constellation of clinical findings can orient the clinician toward a probable diagnostic category prior to imaging. Recognition of these patterns helps avoid inappropriate early surgical exploration and guides selection of biochemical testing and biopsy strategy. Thus, clinical assessment represents the first step in biologically directed evaluation rather than merely a preliminary examination.

The relationship between presenting symptoms and likely diagnostic categories is summarized in Table 1.

**Table 1:** Symptom-based diagnostic orientation Data synthesized from references [7,8,13–15]

Clinical Feature	Likely Etiology	Clinical Implication
Fever, weight loss, night sweats	Lymphoma	Avoid upfront surgery
Episodic hypertension, palpitations	Paraganglioma	Biochemical workup required
Young male with midline mass	Germ cell tumor	Tumor markers before biopsy
Large painless abdominal mass	Sarcoma	Surgical disease likely
Neurological symptoms	Neurogenic tumor	MRI evaluation recommended

Imaging evaluation

Cross-sectional imaging is the cornerstone of evaluation for retroperitoneal masses because clinical examination alone cannot reliably determine organ of origin, tissue composition, or resectability [16]. The primary goal of imaging is not merely to confirm the presence of a mass but to characterize tumor biology, define anatomical relationships, guide biopsy, and anticipate surgical strategy.

Contrast-enhanced computed tomography (CT) is the first-line modality and provides detailed information regarding tumor size, internal architecture, calcification, necrosis, fat content, and relationship to major vessels and adjacent organs [16]. A key feature distinguishing retroperitoneal from intraperitoneal tumors is organ displacement rather than early invasion. For example, kidneys or bowel loops are often pushed aside, indicating extrinsic origin and potential resectability despite large tumor size.

Certain radiologic patterns strongly correlate with histology and assist in diagnostic stratification. Macroscopic fat with thick septations or nodular non-lipomatous components suggests liposarcoma [17]. Homogeneous soft tissue masses that envelop major vessels without causing luminal narrowing are characteristic of lymphoma, reflecting infiltrative but non-destructive growth [13]. Tumors arising from the inferior vena cava or demonstrating intraluminal extension suggest leiomyosarcoma [17]. Well-circumscribed cystic lesions with degenerative change frequently represent schwannoma or other neurogenic tumors [15].

Magnetic resonance imaging (MRI) serves as a complementary modality when neural involvement or vascular invasion is suspected, as it provides superior soft tissue contrast and multiplanar evaluation [15]. MRI is particularly useful for tumors extending into the spinal canal or neural foramina. Positron emission tomography-computed tomography (PET-CT) is not routinely required for primary sarcoma diagnosis but is valuable in staging lymphoma and assessing treatment response [13].

Beyond diagnosis, imaging determines operability. Identification of unresectable disease such as extensive mesenteric root involvement or bilateral major vascular encasement, prevents non-therapeutic laparotomy. Conversely, recognition of organ displacement rather than invasion supports aggressive surgical planning. Thus, imaging functions as a decision-making tool guiding biopsy location, operative approach, and multidisciplinary treatment sequencing rather than a purely descriptive investigation.

Characteristic radiologic patterns that guide diagnostic stratification are summarized in Table 2.

**Table 2:** Imaging clues to tumor biology Data synthesized from references [13,15–17]

Imaging Finding	Probable Diagnosis	Management Direction
Macroscopic fat with septations	Liposarcoma	Surgical planning
Vessel encasement without obstruction	Lymphoma	Biopsy → chemotherapy
Tumor arising from the inferior vena cava	Leiomyosarcoma	Surgical + vascular planning
Well-defined cystic mass	Schwannoma	Usually benign resection
Neural foraminal extension	Neurogenic tumor	MRI characterization

Biopsy strategy

Image-guided tissue diagnosis is a critical step in the modern management of retroperitoneal masses because therapeutic decisions depend primarily on histology rather than tumor size or anatomical location [9]. Historically, many retroperitoneal tumors were explored surgically to establish diagnosis; however, this frequently resulted in non-therapeutic laparotomy in patients with lymphoma or germ cell tumors, exposing them to operative morbidity while delaying definitive treatment [14]. The widespread adoption of percutaneous core needle biopsy has therefore shifted practice toward biology-directed therapy.

Core needle biopsy performed under CT or ultrasound guidance provides adequate tissue for histopathological examination, immunohistochemistry, and molecular analysis in most cases [18]. Multiple cores should be obtained from viable solid components while avoiding necrotic areas, as necrosis may lead to nondiagnostic samples. Fine-needle aspiration alone is generally insufficient because architectural patterns and tumor grading cannot be reliably assessed [19].

A common concern is tumor seeding along the biopsy tract. Contemporary evidence demonstrates that when the needle pathway is planned within a future surgical resection field, the risk of clinically significant tract implantation is extremely low and should not deter biopsy when histology will alter management [18]. For this reason, biopsy planning should involve the surgical team so that the tract can be excised during definitive resection if required.

Biopsy is mandatory when lymphoma, metastatic disease, or germ cell tumor is suspected because these conditions are treated primarily with systemic therapy [13,18]. In contrast, selected tumors with classic imaging features of resectable well-differentiated liposarcoma may proceed directly to surgery, as histology will not change initial management [9]. Nevertheless, even in such cases, many centers still prefer preoperative biopsy to guide multidisciplinary planning.

The biopsy result determines the entire therapeutic pathway. A diagnosis of lymphoma or germ cell tumor directs immediate systemic therapy, whereas confirmation of sarcoma leads to surgical planning and evaluation for neoadjuvant treatment. Thus, biopsy transforms an indeterminate mass into a biologically defined disease and represents the most important decision point before intervention.

The indications for pre-treatment biopsy in retroperitoneal masses are outlined in Table 3.

**Table 3:** Indications for core needle biopsy Data synthesized from references [8,9,13,18–19]

Clinical Scenario	Biopsy Required?	Rationale
Suspected lymphoma	Yes	Systemic therapy required
Germ cell tumor	Yes	Chemotherapy first
Metastatic disease	Yes	Guides oncologic treatment
Unresectable tumor	Yes	Neoadjuvant planning
Classic resectable liposarcoma	May omit	Surgery required regardless

Determining the treatment pathway

The most important clinical decision in the management of retroperitoneal masses is determining whether surgical intervention is appropriate. Unlike many intra-abdominal tumors in which resection is routinely indicated, retroperitoneal lesions represent biologically diverse entities requiring fundamentally different treatment strategies [9]. Therefore, management must be guided by tumor biology rather than anatomical presence alone.

Retroperitoneal sarcomas are predominantly localized diseases in which complete macroscopic resection offers the only realistic chance of durable control [9]. Delay in surgery may allow progressive enlargement, increasing operative complexity and reducing the likelihood of complete resection. Consequently, once sarcoma is confirmed and deemed resectable, operative planning becomes the central component of management.

In contrast, lymphoma is a systemic hematologic malignancy treated primarily with chemotherapy, and surgery has no therapeutic role beyond obtaining tissue diagnosis [13]. Similarly, primary retroperitoneal germ cell tumors are highly chemosensitive and should receive systemic therapy before any surgical intervention [8]. Performing extensive surgery in these conditions not only fails to provide benefit but may also delay curative treatment and increase morbidity.

Another important consideration is resectability. Tumors involving the mesenteric root extensively or encasing multiple critical vascular structures may not be amenable to complete resection. In such cases, neoadjuvant therapy or systemic therapy may be preferable to immediate surgery. Therefore, treatment selection requires integration of histology, imaging, and multidisciplinary assessment.

Failure to distinguish these biological categories can result in inappropriate management - either unnecessary surgery for systemic disease or delayed surgery for resectable sarcoma. The objective is not simply to remove a mass but to select the correct therapy for the disease it represents. Thus, the decision to operate represents a diagnostic conclusion rather than an automatic response to tumor presence [14].

Treatment orientation based on tumor biology is summarized in Table 4.

**Table 4:** Treatment orientation based on tumor biology Data synthesized from references [8,9,13]

Tumor Category	Biological Nature	Initial Treatment Strategy
Retroperitoneal sarcoma	Localized disease [3]	Surgical resection
Lymphoma	Systemic malignancy	Chemotherapy
Germ cell tumor	Highly chemosensitive	Chemotherapy
Metastatic disease	Secondary involvement	Systemic therapy
Benign tumors	Non-invasive behavior	Observation/selective surgery

Major tumor types

Retroperitoneal masses encompass several biologically distinct tumor groups, and understanding subtype-specific behavior is essential because prognosis and management differ substantially between them [9]. Treatment decisions must therefore be individualized according to histology rather than generalized across all retroperitoneal tumors.

Liposarcoma

Liposarcoma is the most common primary retroperitoneal sarcoma and accounts for a significant proportion of cases in large institutional series [9]. Well-differentiated liposarcoma is characterized by slow growth, large tumor size at presentation, and a strong tendency for local recurrence. Despite frequent recurrence, metastatic spread is uncommon in purely well-differentiated tumors [11]. The principal threat to survival is progressive local disease rather than distant metastasis.

Dedifferentiated Liposarcoma

Dedifferentiated liposarcoma, in contrast, contains non-lipogenic high-grade components and exhibits more aggressive behavior, with increased risk of both local recurrence and distant metastasis [11]. Identification of MDM2 amplification assists in confirming diagnosis and distinguishing these tumors from benign lipomatous lesions.

Leiomyosarcoma

Leiomyosarcoma commonly arises from smooth muscle of major vascular structures, particularly the inferior vena cava [11]. These tumors often present with symptoms related to vascular compression or obstruction. Unlike liposarcoma, leiomyosarcoma demonstrates a higher propensity for hematogenous metastasis, particularly to the lungs and liver [11]. Therefore, systemic surveillance plays a greater role in postoperative follow-up for this subtype.

Lymphoma

Retroperitoneal lymphoma usually manifests as bulky nodal masses that envelop major vessels without causing luminal narrowing, a characteristic imaging feature [13]. Because lymphoma represents systemic disease, treatment consists of multi-agent chemotherapy, often combined with immunotherapy depending on subtype. Surgical excision does not improve survival and is generally avoided except for diagnostic biopsy.

Germ Cell Tumors

Primary retroperitoneal germ cell tumors occur predominantly in young males and may arise from aberrant embryologic germ cell migration [8]. These tumors are highly responsive to platinum-based chemotherapy. Measurement of serum tumor markers is essential for diagnosis and monitoring response. Surgical resection is reserved for residual masses after completion of systemic therapy.

Neurogenic Tumors

Schwannomas and other benign nerve sheath tumors are typically slow growing and often asymptomatic until large [15]. In contrast, paragangliomas may secrete catecholamines and present with episodic hypertension and sympathetic symptoms. Preoperative biochemical evaluation and appropriate pharmacologic preparation are mandatory to prevent perioperative cardiovascular complications.

Understanding these distinct biological behaviors allows clinicians to tailor surgical aggressiveness, systemic therapy, and surveillance protocols according to tumor subtype rather than applying uniform management principles to all retroperitoneal masses [20].

Surgical management principles in retroperitoneal sarcoma surgery remains the cornerstone of curative treatment for retroperitoneal sarcoma because disease progression is predominantly locoregional rather than metastatic at presentation [9]. The objective of surgery is not simply tumor removal but durable local disease control, as uncontrolled intra-abdominal recurrence represents the leading cause of death in many patients.

Unlike organ-confined malignancies, retroperitoneal sarcomas arise in a potential space lacking true anatomical boundaries. The apparent tumor capsule represents compressed surrounding tissue rather than a reliable oncologic barrier, and microscopic tumor infiltration commonly extends beyond visible margins [21]. Therefore, tumor enucleation or limited excision is associated with high local recurrence rates and should be avoided.

Modern surgical strategy emphasizes en bloc compartmental resection, in which the tumor is removed together with adjacent structures that form the anatomic compartment of origin [22]. Organs such as kidney, colon, adrenal gland, and psoas fascia may be resected even without macroscopic invasion to eliminate microscopic disease and improve local control. This approach has demonstrated improved oncologic outcomes compared with simple excision in high-volume centers.

Avoidance of tumor rupture is critical because capsular violation leads to peritoneal dissemination and significantly increases recurrence risk [22]. The tumor should be mobilized circumferentially and removed intact rather than debulked. Piecemeal removal, internal decompression, or partial resection compromises oncologic efficacy and should only be considered for palliation.

Preoperative imaging plays a central role in operative planning. Vascular involvement must be anticipated, particularly in tumors abutting or arising from the inferior vena cava, where vascular reconstruction may be required. Multidisciplinary planning allows safe execution of complex resections and reduces intraoperative complications.

Margin assessment differs from other malignancies. Because wide circumferential margins are rarely achievable in the retroperitoneum, the goal is complete macroscopic clearance (R0 or R1 resection) rather than classical wide margins [21]. An R2 resection, leaving gross residual disease, provides minimal survival benefit and is generally avoided in curative settings.

Repeat resection may be considered for isolated local recurrence in selected patients, as prolonged survival can be achieved when disease remains surgically controllable. Thus, surgical management is a longitudinal strategy rather than a single operation, emphasizing planning, technique, and appropriate patient selection.

Key operative oncologic principles in retroperitoneal sarcoma surgery are summarized in Table 5.

**Table 5:** Core surgical principles in retroperitoneal sarcoma Data synthesized from references [21,22]

Principle	Oncologic Rationale	Practical Implication
En bloc resection	Prevent tumor dissemination	Remove tumor intact
Multivisceral resection	Microscopic extension beyond capsule	Remove adjacent organs prophylactically
Avoid tumor rupture	Prevent peritoneal seeding	No piecemeal excision
Preoperative planning	Anticipate vascular involvement	Multidisciplinary surgery
Accept R1 margin	Wide margins rarely achievable	Goal = macroscopic clearance

Role of radiotherapy and systemic therapy

Although surgery is the principal curative modality for localized retroperitoneal sarcoma, the high rate of local recurrence has led to investigation of adjunctive therapies aimed at improving oncologic outcomes [23]. Because microscopic disease may remain despite complete macroscopic resection, radiotherapy has been explored as a strategy to reduce locoregional relapse.

Preoperative (neoadjuvant) radiotherapy offers several theoretical advantages. Delivering radiation before surgery allows improved target delineation, while the tumor displaces radiosensitive bowel away from the radiation field. Additionally, preoperative treatment may reduce tumor viability and facilitate margin control [23]. Prospective trials have evaluated this approach and demonstrated acceptable toxicity profiles, although the magnitude of benefit appears histology-dependent.

Some evidence suggests that patients with liposarcoma, which is characterized predominantly by local recurrence, may derive greater benefit from improved local control strategies compared with subtypes prone to distant metastasis [23]. However, the decision to administer preoperative radiotherapy must consider tumor size, location, expected surgical margins, and institutional expertise.

Postoperative radiotherapy is less commonly employed because resection often results in bowel occupying the tumor bed, increasing the risk of radiation-induced toxicity. Therefore, adjuvant radiation is generally reserved for selected high-risk cases managed at specialized centers.

The role of systemic chemotherapy in retroperitoneal sarcoma remains controversial and is highly dependent on histologic subtype and grade [9]. High-grade leiomyosarcoma and other aggressive sarcomas with significant metastatic potential may benefit from systemic therapy in selected cases. However, routine chemotherapy for low-grade liposarcoma is not recommended because distant metastasis is uncommon and systemic therapy does not significantly alter local recurrence patterns.

In contrast, systemic therapy is the primary treatment modality for lymphoma and germ cell tumors [13,8]. In these diseases, surgery is limited to tissue diagnosis or resection of residual masses following chemotherapy. Prompt initiation of systemic therapy significantly improves outcomes and should not be delayed by extensive surgical intervention.

Overall, adjunctive therapies serve complementary roles in retroperitoneal tumor management. Their use must be individualized based on histologic subtype, tumor grade, resectability, and multidisciplinary consensus.

Pathological evaluation and prognostic factors

Histopathological assessment is central to prognosis and postoperative management in retroperitoneal tumors because outcome is determined more by tumor biology than by tumor size alone [20]. The pathology report should therefore be interpreted not merely as diagnostic confirmation but as a detailed prognostic document that guides surveillance and adjuvant therapy decisions.

The most important parameters include histologic subtype, tumor grade, mitotic activity, percentage of necrosis, and margin status. The FNCLCC (National Federation of Centers for the Fight Against Cancer) grading system stratifies sarcomas into low-, intermediate-, and high-grade categories and correlates strongly with metastatic potential and survival [24].

Histologic subtype predicts recurrence pattern. Well-differentiated liposarcoma demonstrates low metastatic potential but high local recurrence rates, often recurring repeatedly within the retroperitoneum [11]. Dedifferentiated liposarcoma carries a greater risk of both local recurrence and distant spread. Leiomyosarcoma, in contrast, exhibits a higher propensity for hematogenous metastasis, particularly to the lungs and liver, necessitating systemic surveillance [20].

Margin status is another critical prognostic factor. Because wide circumferential margins are rarely achievable in the retroperitoneum, resections are classified as R0 (microscopically negative), R1 (microscopically positive), or R2 (gross residual disease) [21]. Although R1 resection may still provide meaningful disease control, R2 resection is associated with early recurrence and poor survival.

Close communication between the surgeon and the pathologist improves interpretative accuracy. Orientation of surgical specimens and identification of critical margins allow correlation with preoperative imaging and guide future management decisions. Molecular diagnostics further enhance accuracy; for example, MDM2 amplification confirms liposarcoma and distinguishes it from benign lipomatous tumors [11].

Major pathological prognostic parameters and their clinical significance are summarized in Table 6.

**Table 6:** Pathological parameters and clinical significance Data synthesized from references [11,20,21,24] FNCLCC, National Federation of Centers for the Fight Against Cancer

Parameter	Pathological Meaning	Clinical Interpretation
Histologic subtype	Tumor biology	Predict recurrence pattern
FNCLCC grade	Aggressiveness index	Predict metastasis and survival
Margin status (R0/R1/R2)	Completeness of resection	Determines recurrence risk
Mitotic rate	Cellular proliferation	Higher = aggressive tumor
Tumor necrosis	Rapid growth indicator	Poor prognosis marker

Recurrence patterns and surveillance

Patterns of recurrence in retroperitoneal tumors vary significantly according to histologic subtype and grade, and therefore follow-up strategies must be individualized rather than uniform for all patients [25]. Unlike many intra-abdominal malignancies in which distant metastasis predominates, retroperitoneal sarcomas frequently recur locally within the abdominal cavity. Local progression remains the leading cause of mortality, particularly in liposarcoma, where repeated intra-abdominal recurrence may eventually compromise organ function.

Well-differentiated liposarcoma demonstrates a predominantly locoregional recurrence pattern with relatively low metastatic risk [11]. These tumors may recur multiple times over many years, making long-term surveillance essential. Dedifferentiated liposarcoma carries a higher risk of both local recurrence and distant spread. In contrast, leiomyosarcoma more commonly metastasizes hematogenously, especially to the lungs and liver, and therefore requires systemic imaging in addition to abdominal evaluation [20].

The timing of recurrence also varies. High-grade sarcomas often recur within the first three years following resection, whereas low-grade tumors may recur late, even beyond 5 to 10 years after initial treatment [25]. Because late recurrence is common, discontinuation of follow-up after conventional oncologic intervals may lead to missed treatable disease.

Surveillance protocols typically include periodic contrast-enhanced CT imaging of the abdomen, with chest imaging added for subtypes with metastatic potential. Early follow-up is performed at shorter intervals and gradually extended over time. Multidisciplinary review of surveillance imaging allows detection of resectable recurrence, and repeat surgery may prolong survival in selected patients.

Recommended surveillance strategies according to tumor subtype are outlined in Table 7.

**Table 7:** Suggested surveillance strategy after resection Data synthesized from references [11,20,25]

Tumor Type	Primary Risk	Imaging Strategy
Well-differentiated liposarcoma	Local recurrence	Abdominal CT
Dedifferentiated liposarcoma	Local + distant	Abdomen + chest CT
Leiomyosarcoma	Metastasis	Chest + abdominal CT
High-grade sarcoma	Early recurrence	Frequent imaging in the first 3 years
Benign tumors	Minimal	Symptom-based

Multidisciplinary management

Optimal management of retroperitoneal tumors requires coordinated multidisciplinary care because treatment decisions depend on integration of clinical, radiologic, pathological, and surgical information [12]. No single specialty can independently determine the correct therapeutic pathway, and outcomes improve significantly when patients are evaluated within specialized sarcoma teams.

Multidisciplinary tumor boards play a central role in sequencing care. Radiologists define tumor origin and resectability, pathologists confirm histology and grade, surgeons assess operability, and medical and radiation oncologists determine the need for adjunctive therapy. This collaborative process prevents non-therapeutic operations in systemic malignancies and avoids delayed surgery in resectable sarcoma.

Referral to high-volume centers has been associated with improved survival and lower local recurrence rates [12]. These centers provide expertise in complex imaging interpretation, image-guided biopsy planning, multivisceral resection, vascular reconstruction, and postoperative surveillance. In addition, consistent pathological evaluation improves diagnostic accuracy and prognostic stratification.

Multidisciplinary coordination also allows individualized treatment planning. For example, patients with borderline resectable tumors may benefit from neoadjuvant therapy, while those with resectable low-grade tumors may proceed directly to surgery. Similarly, recurrent disease can be assessed for repeat resection rather than immediate systemic therapy in selected cases.

Thus, retroperitoneal tumor management represents a continuum of coordinated decision-making rather than a single operative event. Integration of multiple specialties transforms treatment from procedure-based care to biologically guided oncologic management, improving both safety and long-term outcomes.

Discussion

Retroperitoneal masses represent a distinct clinical entity in which management is determined primarily by tumor biology rather than anatomical presence alone. Advances in cross-sectional imaging and image-guided biopsy have shifted care away from exploratory surgery toward histology-directed therapy, allowing prompt identification of tumors requiring systemic treatment such as lymphoma and germ cell tumors [6].

Once diagnosis is established, treatment pathways diverge significantly. Retroperitoneal sarcomas behave predominantly as locally progressive diseases in which complete macroscopic resection offers the best chance of durable control [3]. This justifies compartmental and multivisceral resection even in the absence of overt organ invasion. In contrast, systemic tumors derive no benefit from aggressive surgery and operative intervention may delay effective medical therapy [9,11].

Close collaboration between the surgeon and the pathologist is equally important. Histologic subtype, grade, and margin status determine recurrence risk and guide surveillance strategy [23]. Consequently, the pathology report functions as a prognostic tool directing multidisciplinary care rather than merely confirming diagnosis.

Overall, management of retroperitoneal masses illustrates the transition from anatomy-based surgery to biology-based treatment, where accurate diagnosis and coordinated decision-making are more important than technical resection alone.

Limitations

This review is narrative in design and relies primarily on retrospective institutional series and consensus guidelines because retroperitoneal tumors are rare and heterogeneous. The absence of large randomized trials limits direct comparison between treatment strategies. In addition, management approaches vary between specialized centers, and treatment decisions must therefore be individualized within a multidisciplinary setting.

## Conclusions

Retroperitoneal masses require a structured diagnostic pathway grounded in tumor biology rather than reflexive operative intervention. Careful interpretation of clinical features, imaging, and image-guided biopsy allows differentiation between localized tumors requiring resection and systemic diseases requiring medical therapy. Retroperitoneal sarcomas remain surgical diseases in which complete macroscopic clearance offers the best chance of durable control, whereas lymphoma and germ cell tumors benefit primarily from systemic therapy.

Optimal outcomes depend on coordinated multidisciplinary management. Integration of radiologic planning, oncologic surgery, and pathological risk stratification allows individualized treatment and appropriate surveillance. A biologically oriented approach therefore minimizes unnecessary surgery while improving oncologic effectiveness in this complex group of tumors.
